# Secondary pulmonary alveolar proteinosis: a single-center retrospective study (a case series and literature review)

**DOI:** 10.1186/s12890-018-0590-z

**Published:** 2018-01-25

**Authors:** Dongmei Zhang, Xinlun Tian, Ruie Feng, Xiaobei Guo, Peng Wang, Yusen Situ, Yi Xiao, Kai-Feng Xu

**Affiliations:** 1 Department of Respiratory Medicine, Peking Union Medical College Hospital, Chinese Academy of Medical Sciences & Peking Union Medical College, Beijing, China; 2 Department of Pathology, Peking Union Medical College Hospital, Chinese Academy of Medical Sciences & Peking Union Medical College, Beijing, China; 3 Laboratory Department, Peking Union Medical College Hospital, Chinese Academy of Medical Sciences & Peking Union Medical College, Beijing, China; 4 Department of Biochemistry, Faculty of Medicine and Dentistry, University of Alberta, Edmonton, Canada; 5 Present address: Department of Respiratory and Critical Care Medicine, Beijing Institute of Respiratory Medicine and Beijing Chaoyang Hospital, Capital Medical University, Beijing, China

**Keywords:** Secondary pulmonary alveolar proteinosis (sPAP), Anti-granulocyte macrophage colony stimulating factor autoantibodies (GM-CSF-Ab), Myelodysplastic syndrome (MDS), Tuberculosis (TB)

## Abstract

**Background:**

Secondary pulmonary alveolar proteinosis (sPAP) is an extremely rare disease. The clinical features of sPAP patients remain to be summarizeds.

**Methods:**

Patients pathologically diagnosed with PAP and with negative results for anti-granulocyte macrophage colony stimulating factor (GM-CSF) autoantibodies from Peking Union Medical College Hospital between January 2000 and July 2016 were retrospectively studied. The PubMed database was also searched for literature to collect published cases.

**Results:**

In our center, nine patients were diagnosed as sPAP with a median age of 37 years. Hematological disorders, including myelodysplastic syndrome (MDS), chronic myelogenous leukemia (CML), and pulmonary tuberculosis (TB) infection were the underlying diseases. Cases secondary to MDS had very poor prognosis as all of them survived less than 2 years after their diagnosis, while those secondary to TB had favorable prognosis. Only 33.3% of cases showed interlobular septal thickening in our sPAP group. Through literature review, 164 sPAP cases were collected. The age at diagnosis was 45.0 ± 14.8 years old and the gender radio was 1.20:1 (M:F). 61.9% of cases were diagnosed by bronchoscopy. MDS and CML were common underlying diseases in 34.1% and 15.2% of patients, respectively. Patients with sPAP secondary to hematological diseases had a short survival time and half of them died within 14.95 months after diagnosis.

**Conclusions:**

MDS and TB infection were the most frequent underlying causes of sPAP in this single-center research in China, with cases secondary to MDS having a poor survival rate. sPAP was more likely to be secondary to hematological disorders, especially MDS and CML and had a fairly poor prognosis in published cases. sPAP should be suspected in PAP patients whose CT scan presents only ground-glass opacities without interlobular septal thickening.

**Electronic supplementary material:**

The online version of this article (10.1186/s12890-018-0590-z) contains supplementary material, which is available to authorized users.

## Background

Pulmonary alveolar proteinosis (PAP), a rare disease with an estimated prevalence of 3.7 to 6.2 cases per million persons [1, 2], is characterized by the accumulation of lipoprotein material in alveoli and terminal respiratory airways [3]. Current knowledge about PAP is based on case studies and individual case reports, and as such, the prevalence of PAP may vary due to differences in medical systems and facilities in different countries. According to the pathogenesis, PAP can be classified in three ways: congenital PAP, secondary PAP (sPAP) and autoimmune PAP (or acquired PAP, or idiopathic PAP) [4]. sPAP mainly develops as a result of hematological diseases, the use of immunosuppression drugs, dust inhalation and certain chronic infections with impaired alveolar macrophage function. Autoimmune PAP, which accounts for over 90% of PAP cases, is an autoimmune disease with autoantibodies targeting granulocyte macrophage colony stimulating factor (GM-CSF) [5].

According to previous reports, sPAP is different from autoimmune PAP in aspects besides the pathogenesis. For example, sPAP has different features on high-resolution computed tomography (HRCT) compared to autoimmune PAP [6] and sPAP has a much poorer prognosis compared with autoimmune PAP [7]. Knowledge of the clinical characteristics of sPAP are still limited as the majority of current literature is in the form of case reports, the largest series being a report by Ishii H which included 40 Japanese patients [7]. As we do not know whether the prevalence of sPAP in China has any differences compared to other countries, we retrospectively collected sPAP patients hospitalized from 2000 to 2016 in our hospital, listed their clinical features and symptoms, and we also reviewed all published cases in literature to learn more about the characteristics of sPAP around the world.

## Methods

### Study design and participants

This is a case study of patients who were diagnosed with PAP in Peking Union Medical College Hospital (PUMCH) between Jan. 1st, 2000 and July 1st, 2016. Medical files were retroactively collected on Feb. 2017. The study was approved by the Ethical Committee of PUMCH (S-K-215), and universal informed consent forms were signed when each subject was admitted to our hospital. The patients or their kin have given written informed consent to publish these case details. Patients had a clinical diagnosis of PAP, which was further confirmed pathologically by testing for amorphous periodic Acid-Schiff (PAS)-positive granules, found either in milky broncho-alveolar lavage fluid (BALF) or in alveolar structures of lung biopsy tissues. Patients under 18 years old were excluded. Serum GM-CSF autoantibodies were evaluated using an enzyme-linked immunosorbent assay, as previously described [8–10]. Patients with positive serum GM-CSF autoantibodies (> 5 μg/mL) were also excluded.

### Methods of data analysis

The patients’ quantitative characteristics such as age at diagnosis were summarized by median and range. The qualitative characteristics such as general appearance, imaging features and other organ complications were presented as a frequency distribution or percentage. Authors had no access to information that could identify individual participants during or after data collection.

## Results

### Demographic characteristics

In total, among 157 hospitalized PAP cases, 9 patients were diagnosed with sPAP. Their clinical characteristics are summarized in Table 1. In these 9 patients, 3 were female and 6 were male, and they ranged in age from 20 to 56 years (median age 37 years). At the time of administration, 5 (5/9) of the cases complained of fever and cough, followed by weakness (4/9), weight loss (3/9), and dyspnea or exertional dyspnea (3/9). Diagnostic procedures included BALF (3/9), CT guided biopsy (2/9), open lung biopsy (2/9), transbronchial lung biopsy (TBLB) plus BALF (1/9), and video-assisted thoracic surgery (VATS) (1/9).

**Table 1:** Characteristics of Patients with secondary pulmonary alveolar proteinosis in Peking Union Medical College Hospital

	Gender	Age	Smoking	Clinical Symptoms	Diagnostic Procedure	Primary Disease	PaO_2_ mmHg
1	Male	38	Current	Weight loss, weakness, night sweats	BALF	MDS-MLD	64.1
2	Female	26	Never	Fever, productive cough	CT guided percutaneous lung biopsy	TB	94.5
3	Male	20	Current	Exertional dyspnea, dry cough	Surgical biopsy	CML	97.4
4	Male	26	Never	Weakness, fever	BALF+TBLB	MDS-SLD	NA
5	Male	37	Never	Dyspnea, dry cough	BALF	MDS-U	48
6	Male	33	Never	Fever, weight loss, weakness	Surgical biopsy	MDS-EB	34.6
7	Male	56	Never	Fever, dry cough	VATS	TB	84.1
8	Female	54	Never	Weight loss,weakness, fever	CT guided percutaneous lung biopsy	TB	39
9	Female	51	Never	Dyspnea, productive cough	BALF	TB	35.6
	CT Scan Features				Comorbidity/Complication	Treatment	Outcomes
1	diffused bilateral GGO				None	WLL, Danazol, Retinoic acid,	Die
2	bilateral GGO patchy geographic pattern, interlobular septal thickening,small scattered nodules				Hepatitis	INH, RIF, EMB, Levofloxacin	Recovery
3	bilateral GGO patchy geographic pattern, bilateral cysts				None	Hydroxyurea	NA
4	diffused bilateral GGO, small nodules				Type B viral hepatitis	Prednisone	Die
5	bilateral GGO with a crazy paving pattern,enlarged mediastinal lymph nodes				*Mycobacterium chelonei*infection	Antibiotics, traditional medicine	Die
6	diffused bilateral GGO, interlobular septal thickening,small scattered nodules				Hospital acquired pneumonia	Danazol, Retinoic acid, WLL	Die
7	bilateral GGO with a crazy paving pattern, interlobular septal thickening				None	INH, RIF, EMB,Clarithromycin, Oxifloxacin	Improved
8	diffused bilateral GGO, small scattered nodules				None	INH, RIF, EMB,Rifapentine	Stable
9	diffused bilateral GGO, septal thickening				Thrombopenia, Sjogren’s syndrome	INH, RIF, EMB,Levofloxacin, WLL	Improved

*BALF* broncho-alverolar lavage fluid, *CML* chronic myelogenousleukemia;*EMB* ethambutol, *GGO* ground-glass opacities, *INH* isonicotinic acid hydrazide, *MDS* myelodysplastic syndrome(SLD:single lineage dysplasia; MLD: multilineage dysplasia; EB: excess blasts; U: unclassifiable), *NA* not available, *RIF* rifampicine, *TB* tuberculosis, *TBLB* transbronchial lung biopsy, *VATS* via video-assisted thoracic surgery, *WLL* whole lung lavage

### Primary diseases

The primary diseases of these 9 sPAP cases were variable, as 5 cases arose from hematologic disorders (4 myelodysplastic syndrome (MDS) and 1 chronic myelogenous leukemia (CML)) and 4 cases from tuberculosis (TB). *Mycobacterium chelonei* infection was detected in one patient with underlying MDS using a subcutaneous nodule biopsy, and another patient was affected by hospital-acquired pneumonia with obscure pathogens, and neither of their sputum nor BALF cultures tested positive.

### CT features

As shown in Table 1, all of our cases presented bilateral ground-glass opacities (GGO) on their CT scans. Among them, GGO with a diffuse pattern was the most frequent pattern, presenting in 5 (5/9) patients, while a patchy geographic pattern and crazy paving pattern each presented in 2 (2/9) patients respectively. Small scattered nodules were found in both TB (2/4) and MDS patients (2/4). The patient infected with *M. chelonei* showed enlarged mediastinal lymph nodes. Interlobular septal thickening, which was thought to be a typical feature of PAP, could only be recognized in three (3/9) of our sPAP patients.

### Pulmonary function tests

At the time of diagnosis, 8 patients underwent arterial blood gas tests and the mean arterial partial oxygen pressure (PaO_2_) was 62.2 mmHg (34.1, 97.4 mmHg) in room air. In 7 patients who performed pulmonary function tests during administration, 6 had normal forced vital capacity (FVC) and forced expiratory volume in the first 1 s (FEV_1_). All 5 cases evaluated for diffusing function showed a remarkable reduction in diffusing capacity for carbon monoxide (DL_CO_) with a mean of 43.1% (22.1%, 58.9%) of the normal predicted value.

### Treatment and prognosis

The median follow-up duration was 4.7 years in these patients. All 4 patients with underlying MDS died within 2 years after diagnosis, and whole lung lavage (WLL) was performed in 2 of them, but symptoms only improved temporarily. Both the symptoms and the CT scans improved in 3 of the 4 patients with TB after anti-TB therapy, and the remaining patient with TB remains stable throughout 8 years of follow-up. Unfortunately, the patient with CML could not be followed-up on. The patient with *M.chelonei* complications (patient 5) had his condition worsen rapidly even with antibiotic administration based on drug sensitivity tests and died 3 months after diagnosis.

### Literature reviews

Texts were searched for using the terms “pulmonary alveolar lipoproteinosis [Title/abstract]” or “pulmonary alveolar proteinosis [Title/abstract]” in the PubMed database. Cases published only in abstract without full-text were excluded. For cases with a history of dust inhalation, only those that tested negative for GM-CSF autoantibodies were included as dust exposure was not a specified factor of sPAP [11].

Using the above search strategy, 1291 articles on PAP were identified. These articles were carefully reviewed to remove reviews, abstracts, commitments, animal researches, relevant pathophysiological studies or pharmaceutical researches, articles about congenital PAP, and articles about autoimmune PAP. We also removed those diagnosed by autopsy post-mortem and pediatric patients less than 18 years old. In the end, 155 cases of sPAP with underlying diseases were found (Additional file 1: Figure S1). In Table 2, we summarized the available characteristics of the 164 cases in total (including our 9 cases).

**Table 2:** Summary of characteristics of 159 secondary pulmonary alveolar proteinosis cases

sPAP	164	Underlying disease	
Age in years	45.0 ± 14.8	Myelodysplastic syndrome	56 (34.1)
Sex ratio (Male: Female)	1.20:1	Chronic myeloid leukemia	25 (15.2)
Clinical symptoms	99	Acute myeloid leukemia	9 (5.5)
Asymptomatic	11 (11.1)	Acute lymphoid leukemia	5 (3.0)
Dyspnea with effort	53 (53.5)	Lymphoma	6 (3.7)
Cough	32 (32.3)	Myelofibrosis	3 (1.8)
fever	38 (38.3)	Other hematological disorders	9 (5.5)
Diagnostic procedure		Infection diseases	13 (7.9)
Bronchoscopy	101 (61.6)	Autoimmune diseases	11 (6.7)
CT guided biopsy	3 (1.8)	Post transplantation	17 (10.4)
VATS	29 (17.7)	non-hematological tumors	5 (3.0)
Surgery lung biopsy	31 (18.9)	Dust inhalation	5 (3.0)

Data is present by number (percentage, %)

*VATS* via video-assisted thoracic surgery

The patients were diagnosed at an age of 45.0 ± 14.8. The male to female ratio was 1.20:1. Bronchoscopy, including BALF and/or TBLB, was the most frequent method used to diagnose sPAP, with 61.9% of cases diagnosed in this manner. Other procedures used included VATS and open lung biopsy, with 17.7% and 18.9% of cases being diagnosed in those manners respectively. Approximately 70% of cases were secondary to hematological diseases, such as MDS (34.1%), CML (15.2%) and AML (acute myelogenous leukemia) (5.5%).

**Fig. 1:**
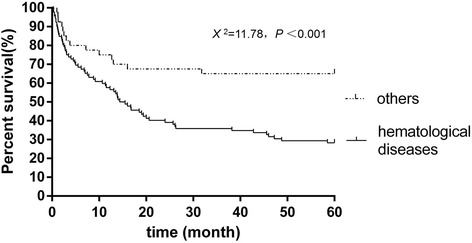
Survival probability of secondary pulmonary alveolar proteinosis cases. In our 9 cases and 155 cases from literatures, 132 cases had the survival information. In 92 cases secondary to hematological diseases, the median survival was 14.95 months as 66 (71.3%) died within 5 years. In 40 cases secondary to other causes, the median survival was to months and 14 (35%) died within 5 years. Log-rank (Mantel-Cox) test *X* ^2^ = 11.78, *P* < 0.001

Underlying infectious diseases, mainly due to TB, non-TB mycobacteria, human immunodeficiency virus, and *Aspergillosis*, consisted of 7.9% of all cases. Autoimmune diseases including Behcet’s disease, vasculitis, Sjogren’s syndrome, and dermatomyositis accounted for 6.7% of the underlying causes. 10.4% of sPAP patients were secondary to lung or kidney transplantation. The other rare underlying diseases were non-hematological tumors and dust inhalation. As shown in Fig. 1, the median survival in 92 cases secondary to hematological diseases was only 14.95 months, which was significantly shorter than sPAP secondary to other causes (*p* < 0.001).

## Discussion

Since sPAP is a rare disease, less than 200 cases have been reported throughout the world, with most of them in form of case reports. H. Ishii [7] summarized the characteristics of 40 Japanese sPAP patients, which was the largest retrospective observational study about sPAP that we found. To the best of our knowledge, our study is the largest series of sPAP from a single center in China. We present some interesting findings. Firstly, TB is also a frequent underlying disease of sPAP in China, and fortunately, after effective anti-TB therapy, sPAP can be improved concurrently. Furthermore, hematologic disorders, especially MDS, are the predominant primary disease of sPAP, and these patients have a terrible prognosis with the majority dying within 2 years after diagnosis. Complications from other infections may accelerate the advancement of the disease.

In our center, 5.73% of PAP cases were determined to be sPAP, which is slightly lower than the percentage of sPAP (8.3% to 10%) in the Japanese case series [6, 7]. Both our study and existing literature illustrates that hematologic disorders are the most frequent underlying disease of sPAP. The study including 31 cases of sPAP with MDS found that sPAP is an important risk factor predicting adverse outcome of MDS with a 2-year survival of 46.2% [12]. In our cases, all of the 4 patients with MDS as the underlying disease died within 2 years. The pathomechanism of sPAP secondary to hematological diseases remains unknown. Mutations in GATA2 may be associated with sPAP [13, 14] since an autosomal dominant familial MDS and AML carried a heritable GATA2 mutation [15] and approximately one third of GATA2 mutation carriers also complicated sPAP [16]. It is speculated that a GATA2 deficiency leads to the alteration of alveolar cellular immunity because these patients had neither GM-CSF receptor mutations nor anti-GM-CSF autoantibodies. In the mice model, T-bet–over expressing T cells act to initiate the pathogenesis of sPAP [17]. Treatment of sPAP should focus on curing the underlying diseases. Bone marrow transplantation or cord blood transplantation can cure sPAP in these patients, but at the cost of an increased risk of infection and mortality [12, 18].

Some infectious diseases such as TB can cause sPAP, possibly due to microorganisms impairing the function of macrophages. The heavy burden of TB in China makes it a significant underlying disease in our cases. In this circumstance, treating the underlying infection(s) can ameliorate sPAP, as shown in our cases, 3 cases improved and 1 case remained stable after anti-TB therapy. However, autoimmune PAP might cause complications with infections including TB, *Nocardia*, and *Aspergillus*. An anti-GM-CSF autoantibody test is effective to distinguish these two situations since it is negative in the former while positive in the later. Besides antibiotics, GM-CSF replacement is ineffective to the former but of benefit to the latter.

In our study, only 33.3% of cases show interlobular septal thickening, which is regarded as a typical feature in the CT scan of autoimmune PAP patients [19]. Additionally, only 22.2% of them present GGO with patchy geographic pattern, which is more common in autoimmune PAP [6]. As a result, sPAP should be suspected in PAP patients whose CT scans only present GGO without interlobular septal thickening or in the absence of a geographic pattern. More detailed evaluation about infectious and hematologic diseases should be performed. Moreover, qualification of anti-GM-CSF-Ab is valuable for the differentiation of autoimmune or secondary PAP.

Several limitations must be stressed with regards to this study. First, since PAP is a rare disease, the value of this study is limited by its small sample size. Our study was also limited in scope in that the underlying disease in our sPAP cases only included hematological diseases and infections, without covering dust inhalation, autoimmune disease or tumors. Secondly, as a retrospective study, some patient characteristics and information are not comprehensive, such as arterial blood gas and lung function. Furthermore, because our hospital is the largest rare disease treatment center in China, patients admitted are usually more complex and serious cases, and as such, we cannot avoid selection bias. Meanwhile, occupational diseases were restrictively diagnosed and treated in designated hospitals in China, so PAP secondary to occupational exposure is difficult to diagnose in our hospital. It is also difficult to compare the result of a single Chinese center with those all over the world. Thus, we conducted a comprehensive review of all the available cases published in available literature to minimize selection bias. Finally, in the review, we did not account for other factors that may affect the variation of sPAP, such as the geographical features of the patients.

## Conclusions

In summary, this study found that MDS and TB infection were the most frequent underlying causes of sPAP in this single center research in China and cases that secondary to MDS had a poor survival rate. However, after taking all the published cases into consideration, hematological disorders, especially MDS and CML, are the major primary disease in 68.9% of cases. sPAP was often middle age onset, but it had a fairly poor prognosis when the underlying diseases were hematologic disorders. The diagnosis of sPAP should be considered in PAP patients whose CT scan only presents GGO without interlobular septal thickening.

## Additional file

Additional file 1: Figure S1. Trial inclusion flow chart. (TIFF 200 kb)

## Abbreviations

AML: Acute myelogenous leukemia
BALF: Bronchoalveolar lavage fluid
CML: Chronic myelogenous leukemia
CT: Computed tomography
DL_CO_: Diffusing capacity for carbon monoxide
FEV_1_: Forced expiratory volume in the first 1 second
FVC: Forced vital capacity
GGO: Ground-glass opacities
GM-CSF-Ab: Granulocyte macrophage colony stimulating factor autoantibodies
HRCT: High-resolution computed tomography
MDS: Myelodysplastic syndrome
PAP: Pulmonary alveolar proteinosis
PAS: Periodic Acid-Schiff
PUMCH: Peking Union Medical College Hospital
sPAP: Secondary pulmonary alveolar proteinosis
TB: Tuberculosis
TBLB: Transbronchial lung biopsy
VATS: Video-assisted thoracic surgery
WLL: Whole lung lavage.
