# Supplementary figures and images for: Secondary pulmonary alveolar proteinosis: a single-center retrospective study (a case series and literature review)
Source: BMC Pulm Med. 2018 Jan 25;18:15. doi: 10.1186/s12890-018-0590-z (PMC5784666; doi:10.1186/s12890-018-0590-z)

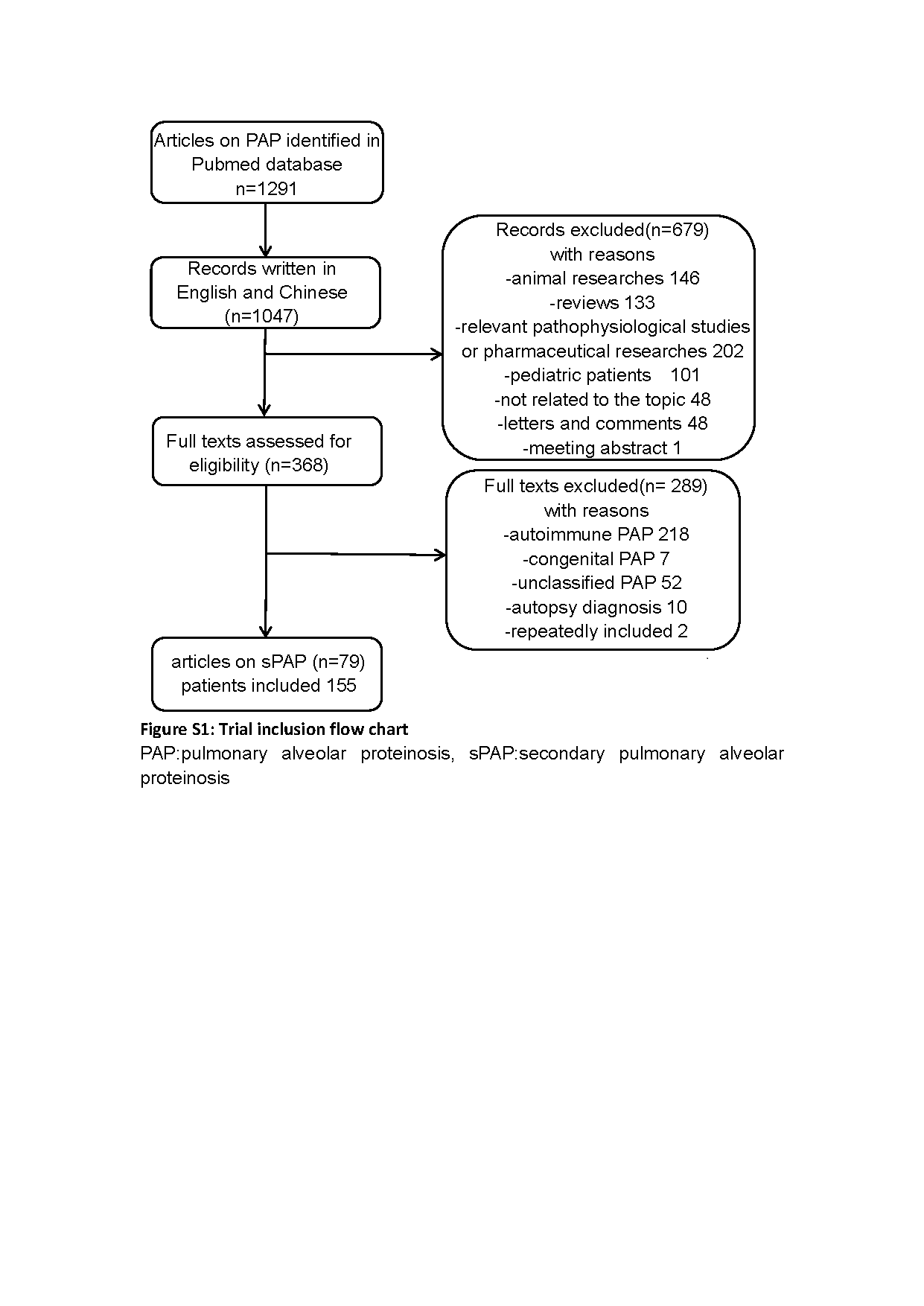

Supplement: Additional file 1: Figure S1. — Trial inclusion flow chart. (TIFF 200 kb) [file 12890_2018_590_MOESM1_ESM.tiff]
